# Closing the treatment gap for mental, neurological and substance use disorders by strengthening existing health care platforms: strategies for delivery and integration of evidence-based interventions

**DOI:** 10.1186/s13033-015-0031-9

**Published:** 2015-12-30

**Authors:** Rahul Shidhaye, Crick Lund, Dan Chisholm

**Affiliations:** Centre for Chronic Conditions and Injuries, Public Health Foundation of India, 19, Rishi Nagar, Char Imli, Bhopal, Madhya Pradesh India; CAPHRI School for Public Health and Primary Care, Maastricht University, Maastricht, The Netherlands; Department of Psychiatry and Mental Health, Alan J Flisher Centre for Public Mental Health, University of Cape Town, Cape Town, South Africa; Centre for Global Mental Health, Institute of Psychiatry, Psychology and Neuroscience, King’s College London, London, UK; Department of Mental Health and Substance Abuse, World Health Organization, Geneva, Switzerland

**Keywords:** Delivery of health care, Health systems research, Health services research, Mental disorders, Low and middle income countries, Integrated care

## Abstract

This paper outlines the main elements and features of a mental health care delivery platform and its delivery channels. These include evidence-based interventions that can be delivered via this platform as well as broader health system strengthening strategies for more effective and efficient delivery of services. The focus is broadly on health systems perspective rather than strictly disorder-oriented intervention analysis. A set of evidence-based interventions within the WHO pyramid framework of self-care, primary care, and specialist care have been identified; the main challenge lies in the translation of that evidence into practice. The delivery of these interventions requires an approach that puts into practice key principles of public health, adopts systems thinking, promotes whole-of-government involvement and is focused on quality improvement. Key strategies for effective translation of evidence into action include collaborative stepped care, strengthening human resources, and integrating mental health into general health care. In order to pursue these principles and strategies using a platform-wide approach, policy makers need to engage with a wide range of stakeholders and make use of the best available evidence in a transparent manner.

## Background

A large proportion of persons affected by mental, neurological and substance use (MNS) disorders do not have access to a wide variety of evidence-based interventions which can prevent and treat these disorders, resulting in a huge treatment gap [1]. This problem is not just limited to MNS disorders as cost-effective interventions in other health sectors are inadequately provided and underused [2]. De Savigny and Adams have mentioned, “evidence-based interventions often fail to achieve their goal, not so much due to the inherent flaw in the interventions themselves, but due to the unpredictable behavior of the system around them [2]”. Multiple barriers related to human resources, infrastructure, information and service provision, people’s participation, knowledge, perception of services, help-seeking behavior and overall stewardship and governance related issues affect health system performance [2]. As the effectiveness of MNS disorders is largely determined by the health systems in which they are nested, it is essential to shift focus from a strictly disorder-oriented or ‘vertical’ perspective to a more health systems strengthening or ‘horizontal’ approach. There is also a strong evidence-base supporting common environmental risk factors (such as unhealthy lifestyles) leading to mental and physical non-communicable diseases, often presenting as co-morbidities and treatments for one condition may have side effects that increase the risk of another condition [3]. There are several other reasons for integrating MNS care into general health care systems, including the limited number of specialist healthcare providers, reduced mental health stigma from receiving care in general healthcare services and improved efficiencies.

In the real-world setting, implementation of evidence-based interventions for MNS disorders seldom occurs through the delivery of single vertical interventions, rather these interventions are delivered via so-called platforms—the level of the health or welfare system at which interventions or packages can be most appropriately, effectively, and efficiently delivered [4].

This paper seeks to identify evidence-based interventions that can be appropriately packaged for one or more specific MNS disorders, as well as for different levels or platforms of the health or welfare system. A particular platform is defined based on WHERE the intervention will be delivered (the setting) and WHO will deliver the intervention (the service provider). A specific delivery channel such as a school or a primary health care centre represents the vehicle for the delivery of a particular intervention on a specified platform. Identifying the set of interventions that fall within a particular platform or delivery channel will help decision makers to identify potential opportunities, synergies, and efficiencies. In addition, it is to these delivery platforms or channels that resources are often allocated in practice, for example, to schools or primary health care services, rather than to specific interventions or disorders.

In this paper we outline the main elements and features of a MNS care delivery platform and its delivery channels, such as self-care informal health care, primary health care, or specialized services. We consider evidence-based interventions that can be delivered in general health care settings and MNS care settings, as well as broader health system strengthening strategies for more effective and efficient delivery of services on this platform.

Evidence-based interventions that can also be delivered via population or community platforms—ranging from legislative and regulatory measures aimed at restricting access to means of self-harm/suicide and reducing demand for alcohol to parenting programs during infancy or socio-emotional learning programs for vulnerable—have also been identified and are reported elsewhere [5].

## Elements of a mental health care delivery platform

**Fig. 1:**
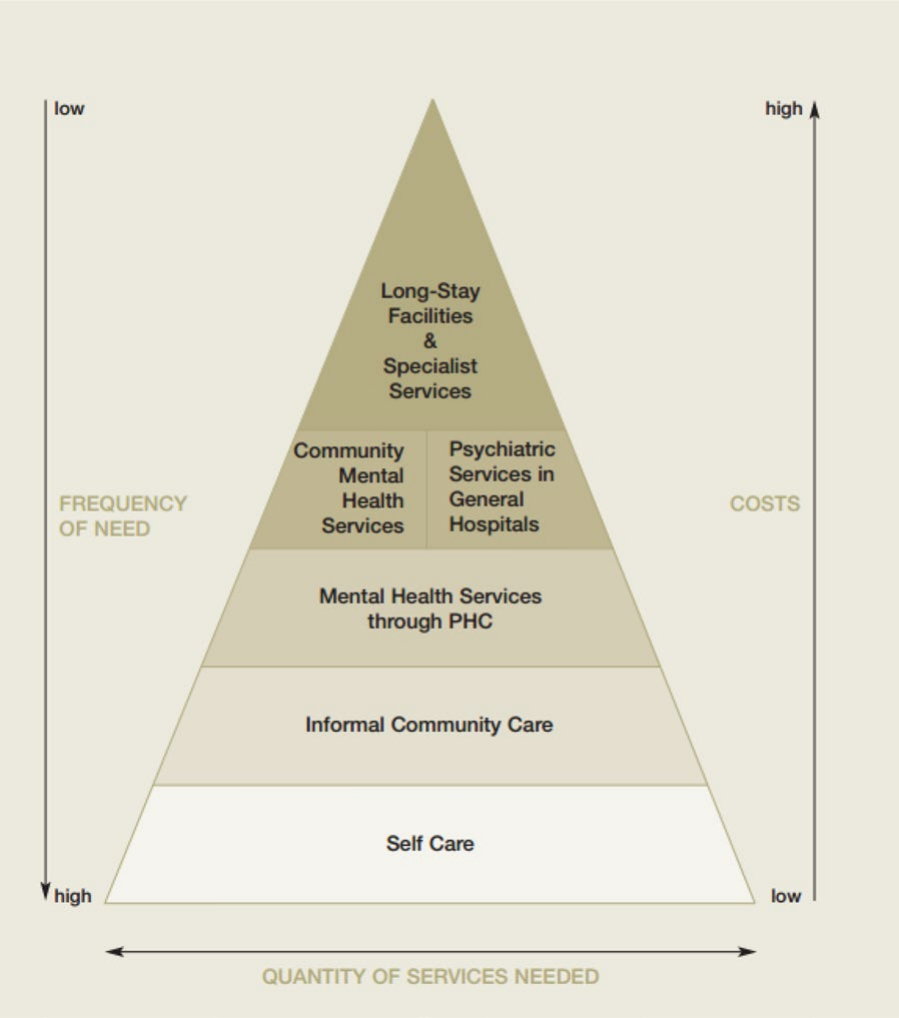
World Health Organization service organization pyramid for an optimal mix of mental health services*. Source*: [Organization of Services for Mental Health: Mental Health Policy and Service Guidance Package. Geneva: WHO, 2003.]

Health care services as a delivery platform for improving population mental health comprise three interlinked service delivery channels: self-care and informal health care; primary health care; and specialist health care. These three key delivery channels map well onto the commonly cited *Service Organization Pyramid for an Optimal Mix of Services for Mental Health* by the World Health Organization (WHO) [6] (Fig. 1).

At each subsequent level of the pyramid the mental health needs of individuals become greater and require more intensive professional assistance, usually resulting in higher costs of care.

### Self-care and informal health care

The foundation of the health care delivery platform rests on self-care and emphasizes health worker–patient partnerships. Persons with MNS disorders and psychosocial disabilities, and their family and friends, play a central role in the management of the mental health problems. The role of individuals may range from collaborative decision-making concerning their treatment, to actively adhering to prescribed medication, to changing health-related behaviors, such as drug and alcohol use, self-management of stress and identification of seizure triggers. Informal health care comprises service providers who are not part of the formal health care system, such as traditional healers, village elders, faith-based organizations, peers, user and family associations, and lay people [6]. Traditional healers are of particular significance as populations throughout East Asia and Pacific, South Asia, Latin America and the Caribbean, and Sub-Saharan Africa often use traditional medicine to meet their health needs [7]. Peers are a key human resource at this level of health care. Peer-led education and behavioral interventions have been effective for a number of target populations with health issues in LMICs [8–10]. Mental health self-help groups form another key component of informal community care. Mental health self-help Groups may be defined as, “any mutual support oriented initiative directed by people with MNS disorders or their family members” [11]. However, informal community care should not be viewed as a substitute for publicly funded, evidence-based mental health care. One of the proximal determinants of help seeking by individuals is the perceived need for care which in turn is dependent on various socio-cultural factors as well as knowledge, attitudes and values that people have towards the health care system [12]. ‘Mental health literacy’ refers to people’s knowledge and beliefs about MNS disorders which aid their recognition, management and help seeking choices. Effective awareness-raising campaigns can result in increased presentation of persons with mental health disorders to primary health care and improved service utilization [13]. In addition to this it is important to develop locally valid ways of understanding, communicating and augmenting perceived needs of people especially with common mental disorders and substance abuse as it has been observed that primary care services have difficulty in identifying and engaging patients with these disorders compared to severe mental disorders. It is also important for clinicians to develop their understanding of local idioms of distress, in order to provide culturally appropriate care.

### Primary health care

Delivery of services for MNS disorders through primary health care is a fundamental component of a health care delivery platform, since it serves as the first level of care within the formal health care system. The strong emphasis on primary health care is due to the fact that the services provided at this level of the health system are generally accessible, affordable, and acceptable for individuals, families, and communities [6]. Where the provision of mental health care is integrated into these services, access is improved, MNS disorders are more likely to be identified and treated, and comorbid physical and mental health problems can be managed more seamlessly.

### Specialist health care

#### Psychiatric services in general hospitals and community mental health services

People with severe MNS disorders may require hospitalization at some point. First-level hospitals (typically at the district level) provide an accessible and acceptable location for 24-h medical care for people with acute worsening of disorders, in the same way that these facilities manage acute exacerbations of physical health conditions [6].

In addition, there is a need for specialist mental health services in the community for severe cases that cannot be managed by generalists. Examples include assertive community treatment teams and community outreach teams, which provide support to service users to enable them to continue to function in the community without requiring admission, and close liaison with general primary care services and other social and criminal justice services [6].

#### Extended-stay facilities and specialist psychiatric services

A small minority of people with MNS disorders will require specialist care beyond that provided in first-level hospitals [6]. For example, people with treatment-resistant or complex presentations may need to be referred to specialized centers for further testing and treatment. Others may occasionally require ongoing care in community-based residential facilities due to their severe mental disorders or intellectual disabilities and lack of family support. Forensic psychiatry is another type of specialist service in this category. The need for referral to specialist and extended-stay services is reduced when general hospitals are staffed with highly specialized health workers, such as psychiatrists and psychologists.

#### Relationships between different delivery channels

No single service delivery channel can meet all mental health needs. For example, primary mental health care must, on the one hand, be complemented by specialist care services to whom primary health workers can turn for referrals, support, and supervision; on the other hand, primary mental health care needs to promote and support self-care and informal community care that encourages the involvement of people in their own recovery. Support of self-care and management can be provided via routine primary care visits or via group sessions led by health or lay workers in health care settings or community venues. In short, the potential of the health care system as a delivery platform for enhanced mental health and well-being can only be fully realized if genuine continuity and collaboration of care occur across the three service delivery channels; the continuity and collaboration, in turn, rely on an appropriate flow of support, supervision, information-sharing and education.

## Evidence-based interventions for health care delivery platforms

A strong evidence base supports integrated services across the different delivery channels of the health care platform. This evidence has been synthesized in a number of publications, including the *mhGAP Intervention Guide* [14], a series of papers on packages of care for MNS disorders in LMIC, published in *PLoS Medicine* [15], and a WHO-WONCA report on mental health in primary health care [16].

For each of the delivery channels, interventions may be categorized as follows:

- Promotion and primary prevention
- Identification and case detection
- Treatment, care, and rehabilitation.

Table 1 summarizes the evidence base for interventions.

**Table 1:** Examples of evidence-based interventions relating to the health care delivery platform by various delivery channels

Delivery channel	Promotion and primary prevention	Identification and case detection	Treatment, care, and rehabilitation
Self-care and informal health care	Adoption of a healthy lifestyle, including diet and physical activity relaxation training Self-monitoring of high risk behaviors, such as substance abuse	Self-detection of depression and anxiety disorders	Web-based psychological therapy for depression and anxiety disorders Self-managed treatment of migraine Self-identification and management of seizure triggers Improving adherence to anti-epileptic treatment by intensive reminders and implementation intention interventions
Primary health care	Parent skills training for internalizing and externalizing problems in child and parental mental health	Screening for developmental delays in children Screening and brief interventions for alcohol use disorders by trained primary health care staff Community-based case finding of psychosis and severe depression Diagnosis of depression, anxiety disorders, maternal depression, alcohol use disorders, dementia, headaches, and epilepsy	Management—pharmacological and psychosocial interventions—of depression, anxiety, psychosis, bipolar disorder, alcohol use disorders, epilepsy, dementia, and drug use based on mhGAP Intervention Guidelines Psychological treatment for depression, anxiety, ADHD, disruptive behaviour disorders in children Cognitive behavioral therapy-based interventions for depression and anxiety disorders in adults and mothers in perinatal period Management of alcohol withdrawal in conjunction with motivational interviewing and motivation enhancement involving family and friends Interventions for caregivers of patients with psychosis and dementia Improve quality of antenatal and perinatal care to reduce risk factors associated with intellectual disability Primary healthcare packages for underlying MNS disorders (for suicide and self-harm) Planned follow-up and monitoring of suicide attempters Emergency management of poisoning
Specialist health care		Diagnosis of complex childhood mental disorders Diagnosis of severe psychosis and depression Diagnosis of secondary causes of headache Screening of new-born babies for modifiable risk factors for intellectual disability	Electroconvulsive therapy for severe refractory depression Surgical interventions for refractory epilepsy Pharmacological management of dementia (cholinesterase inhibitors and memantine) Methadone maintenance therapy for opioid dependence, buprenorphine as opioid substitution therapy Management of refractory psychosis using clozapine Management of severe alcohol dependence (along with withdrawal) Management of severe maternal depression using antidepressants Stimulant medication for severe cases of attention deficit hyperactivity disorder Cognitive behavioral therapy based interventions and anger control training for adolescents with disruptive behavioral disorders

## System strengthening strategies for integrated health care delivery

The availability of evidence-based interventions does not ensure their translation into practice. It is critical to address the question of how to integrate evidence-based mental health care interventions into primary care and self-care delivery channels and how to link this integration to specialist care. A comprehensive and multifaceted approach that contains the following elements is essential for the successful integration of mental health into health care systems:

- A *whole-of government approach* involves the promotion, pursuit, and protection of health through concerted action by many sectors of government. These include ministries of planning and development, finance, law and justice, labor, education, and social welfare. The health system cannot tackle the health, social, and economic determinants and consequences of MNS disorders alone.
- A *public health approach* stresses the establishment of partnerships between patient and service providers, as well as equitable access for the whole population [17]. This approach requires the integration of care at the patient level. Services should be person-centered and coordinated across diseases and settings. Collaborative, coordinated, and continuing care, within a framework of evidence-based interventions, provides the foundation of the public health approach. This means providing good-quality, accessible services to those in need, as well as preventing the onset of disease and promoting mental health and well-being over the entire life course [18].
- A *systems approach* to integrated service planning and development encompasses the critical ingredients of a health system—good governance, appropriate resourcing, timely information, as well as the actual delivery of health services or technologies—that need to be in place for desired health outcomes or program goals to be realized. Effective governance, strong leadership, and cogent policy-making merit particular mention, since they provide the framework for appropriate action and subsequent service development. Indeed, a well-articulated mental health policy, along with a clear mental health implementation plan and budget, is a strong driver for change and can appreciably boost efforts to deliver mental health services at primary care level [16].

It is also imperative to understand that ‘context’ in the form of local health system and social influences are inextricably tied up with the outcomes of service delivery changes. Literature from high-income countries suggests that the interventions that work in initial studies lose their effectiveness as they are implemented widely [19]. The effectiveness of an intervention is often based on studies in a small number of settings and the full range of complexity of the intervention may not be fully understood, ultimately resulting in the intervention working in only 50 % of replication sites, implying an equal chance that it will or will not work [20]. Many health systems lack the capacity to integrate new evidence-based interventions and when such systems are not well understood, even the simplest intervention can fail [2]. Nested within the wider health systems strengthening approach, we describe a number of specific strategies for integrated mental health care delivery, but is should be borne that they are context-specific and may not be generalizable in all settings. Nevertheless, the learnings from the relevant literature can be applied after suitable contextual adaptation.

### Strategy 1. Improving the organization and delivery of services through collaborative stepped care

*Collaborative care* is an evidence-based approach to improve the management of MNS disorders at the primary care level. The overall aim of collaborative care is to enhance the quality of care and quality of life, consumer satisfaction, and system efficiency of patients with complex, long-term problems [21]. Collaborative care has been used successfully for the management of common mental disorders such as depression, as well as for comorbidities cutting across multiple services, providers, and settings [22]. Collaborative care is closely related to a stepped care approach; some programs describe themselves as *collaborative stepped care*, in that they incorporate aspects of each approach within their interventions [23]. In the stepped care approach, patients typically start treatment with low-intensity, low-cost interventions. Treatment results are monitored systematically, and patients move to a higher-intensity treatment only if necessary. Programs seek to maximize efficiency by deploying available human resources according to need, reserving the most specialized and intensive resources for those with the most complex or severe problems.

The essential element of collaborative care is a multidisciplinary team approach that seeks to integrate primary care professionals and specialists. Collaborative care rests primarily on the presence of a case manager with enhanced responsibilities for integration of care across comorbid conditions. It starts with systematic identification of those in need, followed by close involvement of patients in joint decision-making regarding their care. It continues with the design of a holistic care plan that includes medication management and psychological interventions, and where appropriate, social care, with a streamlined referral pathway that allows patients to move easily from one service to another. There is provision for regular and planned monitoring of patients and systematic caseload reviews and consultation with mental health specialists regarding patients who do not show clinical improvement [24].

Collaborative care is the best-evaluated model for treating common mental disorders in primary care. A recent Cochrane Collaboration review of 79 randomized controlled trials concluded that collaborative care for depression is consistently more effective than usual care; it has also been shown to be effective in a range of MNS disorders—anxiety disorders, post-traumatic stress disorder—and for improving general health outcomes. The evidence base for collaborative care is mostly from high-income countries (HICs), although evidence from LMICs is growing [25]. The MANAS study in Goa, India, showed that a lay counselor-led collaborative stepped care intervention for depression and anxiety disorders in primary health care settings led to substantial reductions in the prevalence of these disorders, suicidal behaviors, and days of work lost, compared with usual care [23]. The Home Care Program for the elderly people affected by dementia, showed benefits in reducing the caregiver burden and improving caregiver mental health in India [26]. In Chile, a multicomponent intervention lasting 3 months and comprising nine weekly sessions of psycho-educational groups, structured and systematic follow-up, and pharmacotherapy for women with severe depression, led by nonmedical health workers, demonstrated that at 6-months’ follow-up, 70 percent of the stepped care group had recovered, compared with 30 percent in the usual-care group [27]. The program is being rolled out across Chile. A similar program subsequently tested among low-income mothers in postnatal primary-care clinics in Santiago, Chile, demonstrated significant improvement in the intervention group [28].

These case studies described primarily focused on evidence generation and were conducted in a controlled setting. There are also several other case studies from a number of LMICs which demonstrate real-world implementation of this evidence-base. In the city of Sobral, Brazil, primary care practitioners conducted physical and mental health assessments for all patients as part of integrated primary care for mental health. Joint consultations are undertaken among mental health specialists, primary care practitioners, and patients. This model ensures good-quality mental health care, and it serves as a training and supervision tool whereby primary care practitioners gain skills that enable greater competence and autonomy in managing mental disorders [16]. A similar model is being practiced as part of the District Mental Health Programme in Thiruvananthapuram district, Kerala, India. Over time, the primary care centers have assumed responsibility for independently operating mental health clinics with minimal support from the mental health team [16]. The European Headache Federation and Lifting the Burden: the Global Campaign against Headache [29] has proposed a collaborative care model for the management of headache disorders. In this model, 90 percent of people consulting for migraine and tension-type headache can be diagnosed and managed by staff at the primary care level. In case of remaining 10 percent of the patients, common primary and secondary headache disorders can be recognized but not necessarily managed and then these can be referred to the next level, where physicians can provide more advanced care. Finally, specialists can provide advanced care to approximately 1 percent of patients first seen at the first-level and second-level facilities and can focus on the diagnosis and management of the underlying causes of all secondary headache disorders. A demonstration project based on this model is in Yekaterinburg, Sverdlovsk Oblast, Russian Federation [30], and headache services in China have been designed on this model [31].

The collaborative stepped care approach relies heavily on the introduction of additional human resources, identification of core competencies, adequate training to ensure that these core competencies are fulfilled, and specialist support to maintain these competencies.

### Strategy 2. Strengthening human resources for mental health through task sharing

One of the main reasons for the substantial treatment gap for MNS disorders is the lack of a skilled workforce. In HICs, the number of mental health workers is often inadequate; in LMICs, the situation is dramatically worse, with an estimated shortage of 1.18 million workers [32]. The collaborative stepped care approach can be implemented only if skilled human resources are available at the different levels of service delivery.

#### Task-sharing approach

*Task*-*sharing* is a human resource innovation in which the skills to deliver specific mental health care tasks are transferred to appropriately trained and supervised general health workers. This process helps in improving access to evidence-based mental health care and leads to more efficient use of these limited resources. This approach has been evaluated for mental health service delivery, and its efficacy has been established using rigorous evaluation methodologies [23, 27, 33]. Task-sharing is implemented through a collaborative care framework with four key human resources: the community health worker/case manager; the person with a mental health problem and family members; the primary or general health care physician; and the mental health professional [34]. The overall shortage of human resources can be addressed by introducing newly skilled non-specialist health workers at community level; reorienting medical officers and paramedical staff to integrate mental health interventions; and redefining the role of specialists from service provision to leadership, training, and supervision of mental health programs.

The task-sharing approach is at the heart of establishing the collaborative stepped care models; the most crucial element in this approach is the availability of a case manager. Several global case studies have found that primary care for mental health is usually most effective where a mental health coordinator/case manager is responsible for overseeing integration [16]. These case managers can play a crucial role in screening; engaging; educating patients and family members; maintaining close follow-up; tracking adherence and clinical outcomes; and delivering targeted, evidence-based, psychological interventions, such as motivational interviewing, behavioral activation, problem-solving, or interpersonal therapy [35]. The case managers can serve as the link between the primary care and self-care platform and can work under the close supervision of the medical officers.

#### Competency-based and continuing education

Primary care workers function best when their tasks related to mental health service delivery are limited and achievable. The most common reasons for failure to integrate mental health care into primary care programs are the lack of adequate assessment and overly ambitious target setting without the necessary customization of the detailed activities, and a full and explicit agreement on the targets and activities needed to achieve them [35]. A shift away from knowledge-based education to competency-based education is needed. This approach mainly focuses on the skills of providers, with the ultimate goal of improving patient outcomes. *Competency* is defined as an attribute of an individual human resource and the ability of that worker to deliver an intervention to a desired performance standard based on the acquired knowledge and skills. The Institute of Medicine’s (IOM) Forum on Neuroscience and Nervous System Disorders have identified core competencies that specialized and nonspecialized primary care providers might need to help ensure the effective delivery of services for depression, psychosis, epilepsy, and alcohol use disorders in sub-Saharan Africa [36].

Pre-service and in-service training of primary care workers on mental health issues is an essential prerequisite for the integration of mental health into primary care platforms. The training, to the extent possible, should happen in primary care or community mental health care facilities, to ensure that practical experience is gained and that ongoing training and support are facilitated [16]. The effects of training are nearly always short-lived if health workers do not practice newly learned skills and receive ongoing specialist supervision. A trial from Kenya did not find any impact of the training program of medical officers on improvement in diagnostic rates of mental disorders [37]. A quasi-experimental study from Brazil had similar findings and noted that wider changes in the system of care may be required to augment training and encourage reliable changes in clinical practice [38]. Ongoing support and supervision from mental health specialists are essential. Case studies from Australia, Brazil, and South Africa have demonstrated that a collaborative stepped care approach in which joint consultations and interventions occur between primary care workers and mental health specialists increases the skills of primary care workers and builds mental health networks [16]. It is absolutely imperative to sustain the competencies of the primary care workers and new information technology enabled platforms such as skype and social media applications such as Facebook and Whatsapp can be potentially used for online distant supervision by the specialist. In addition to this decision support algorithms enabled by mobile health, cloud-based electronic health records that can be accessed and updated by any provider, automated medication and appointment reminders offer new opportunities to address systemic barriers to improving coverage of service by the trained human resource [3].

#### Specialist transitioning

Specialists, especially in LMICs, are usually engaged in service delivery. It is imperative to make a transition from providing clinical services to training and supervising the primary health care staff, and providing direct clinical interventions judiciously and sparingly. In two projects focusing on integrated primary care for mental health in city of Sobral, Brazil, and Sembabule district of Uganda, specialists visited primary care settings and assessed patients together with medical officers in primary care. Over time, psychiatrists started taking less active roles while general practitioners assumed added responsibilities, under the supervision of the psychiatrists. Specialists can interact with primary care staff via referral and back-referral [16].

#### Planning and consultation

Involving primary health care staff in overall program planning and rollout process enhances ownership and commitment to achieve the planned outcomes within agreed timelines [35]. Consultations with general practitioners was demonstrated to be one of the key factors in success of the new mental health services in Australia [16]. Decisions must be made after careful consideration of local circumstances; this requires consultation with policy makers, as well as users of mental health services and the families and the primary care staff.

#### Psychotropic medications

It is important to ensure that primary care staff members have the appropriate permission to prescribe psychotropic medications, and they must be adequately trained to perform this task. In many countries, nurses and even general physicians are not permitted to prescribe psychotropic medications. If access to psychotropic medications is to be improved, then initiatives to allow primary care nurses to prescribe psychotropic medications need to be promoted and undertaken, provided appropriate training and supervision is conducted. In Belize, psychiatric nurse practitioners have been given additional prescription rights. In Uganda, general primary care nurses are permitted to prescribe psychotropic medication to patients who require continued medication on the recommendation of a mental health professional [16].

### Strategy 3. Integrating mental health into existing health care delivery channels

Expansion and integration of mental health services in primary health care can be achieved by using existing service delivery for maternal and child health, non-communicable diseases, and HIV/AIDS and tuberculosis [39].

#### Maternal and child health programs

Promising evidence suggests the benefits of the integration of maternal mental health into maternal and child health (MCH) programs [40]. The Thinking Healthy Programme in Pakistan is a simple and culturally appropriate intervention for integrating depression care in a MCH program. The intervention is child-centered, ensuring buy-in from the families and avoiding stigmatization. It is woven into the routine work of the community health workers, so it is not seen as an extra burden but supports the routine work. The Thinking Healthy Programme has been further adapted so that it can be used universally for all women rather than only depressed women [40].

The Perinatal Mental Health Project in the Western Cape Province of South Africa developed a stepped care intervention for maternal mental health that is integrated into antenatal care in three primary care midwife obstetric units [41]. This case study clearly demonstrates that onsite, integrated mental health services increase can access for women who have scarce resources and competing health, family, and economic priorities [41].

Parenting skills training aims to enhance and support the parental role through education and skills enhancement, thereby improving the emotional and behavioral outcomes for children. Primary health care workers can play a significant role in this training. The use of scarce professional resources to train parents is a cost-effective use of resources and can be integrated in primary care services. Several systematic reviews have shown parent skills training to be effective for reducing both internalizing and externalizing problems in children [42, 43], as well as reducing the risk of unintentional childhood injuries [44] and improving the mental health of parents [45].

#### Noncommunicable disease programs

Existing service delivery platforms for noncommunicable diseases are also promising entry points for the integration of mental health into primary care. The collaborative care models discussed demonstrate a strong evidence base for integration in primary care settings. In North America, TEAMcare, US, and TEAMcare, Canada provide team-based primary care for diabetes, coronary heart disease and depression. About 1400 people have received TEAMcare, with a trial showing improvements in medical disease control and depression symptoms [46]. In the United Kingdom, 3 Dimensions of Care for Diabetes (3DFD) uses a team consisting of a psychiatrist and a social worker from an nongovernmental organization embedded in the diabetes care team to integrate medical, psychological, and social care for people with diabetes and mental health problems, and/or social problems, such as housing and debt [47]. The National Depression Treatment Programme in Chile integrated depression care with more traditional primary care programs for the management of hypertension and diabetes within a network of 520 primary care clinics [48]. In Myanmar and in several LMICs, epilepsy has been included as part of the process of local adaptation and implementation of the WHO’s package of essential noncommunicable disease interventions in primary care [24].

#### HIV/AIDS and tuberculosis programs

The WHO’s Integrated Management of Adult and Adolescent Illness (IMAI) is a broadly disseminated health care strategy that addresses the overall health of patients with HIV/AIDS and co-occurring tuberculosis; clear opportunities exist for integration of mental health in this program [49]. In South Africa, the government has published integrated guidelines for all primary health workers, including HIV/AIDS; major non-communicable diseases; and a range of mental health problems, including depression, anxiety, mania, substance abuse, and psychosis. This guideline, called Primary Care 101 (PC101) [50], is used by the national Department of Health as part of a primary care revitalization program to deliver integrated care within a chronic disease management framework [51].

## Resource estimation

In order to achieve the successful and sustainable scale-up of effective interventions and innovative service-delivery strategies mentioned above, it is critical to conduct an analysis of resource estimation (financial as well as human) required to operationalize components of collaborative care model, build and sustain the competencies of the human resources and engage with all the key stakeholders including users and community members. This exercise is beyond the scope of this paper, but has been carried out as part of the Disease Control Priorities 3rd edition [4] and for five low and middle-income countries in the PRogramme for Improving Mental health carE (PRIME) [52].

## Quality of care for MNS disorders

Despite the strong and growing knowledge base for delivery of mental health services, the ‘treatment gap’ for MNS disorders remains unacceptably large, with over 90 % of people with mental disorders in LAMICs going without treatment [53]. This ‘treatment gap’ is not just a quantitative phenomenon; it also contains an important ‘quality’ of care dimension. There is a significant gap between what is known about effective treatment and what is actually provided to and experienced by consumers in routine care [54]. Quality in health care has been defined by the Institute of Medicine as ‘the degree to which health care services for individuals and populations increase the likelihood of desired health outcomes and are consistent with current professional knowledge’ [55]. Good quality care is (or should be) effective, efficient, equitable, timely, person-centered, safe and delivers a positive patient experience [55]. In the language of Universal Health Coverage, it is the difference between contact coverage and effective coverage; that is, substantial improvement in access to care need to be also accompanied by improvement in the quality of service delivery.

Inadequacy of resources and low priority given to the MNS disorders might lead one to think that consideration of the ‘quality’ of care be subservient to the quantity of available and accessible services. However, quality improvement mechanisms ensure that available resources are well utilized—in the sense that those in contact with services actually derive appropriate benefit from evidence-based interventions. Moreover, good quality services help to build people’s confidence in making use of mental health care interventions, thereby increasing the likelihood of seeking the care that they need [56]. Low quality services, on the other hand, lead people with MNS disorders to experience human rights violations and discrimination in health-care settings. In many countries, the quality of care in both inpatient and outpatient facilities is poor or even harmful and can actively hinder recovery [57]. Quality improvement frameworks and guidelines for LAMICs have been developed in the form of a WHO guidance package for quality improvement in mental health services [58]. It provides an integrated resource for the planning and refining of mental health systems on a national scale [56].

## Conclusions

The key points for effective and efficient delivery of MNS services are as follows:

- To deliver interventions for MNS disorders, the focus needs to move from vertical programs to horizontal health service platforms.
- The WHO pyramid framework of self-care, primary care, and specialist care continues to provide a useful approach for understanding potential delivery channels.
- A set of evidence-based interventions within this framework can be identified for promotion/prevention, identification/case detection, and treatment/care/rehabilitation interventions.
- The delivery of these interventions requires an approach that embraces public health, systems, and whole of government principles.
- The key strategies for this delivery are implementing collaborative stepped care, strengthening human resources, and integrating mental health into general health care.
- Finally, it is not only important to improve access to health services for MNS disorders but also to focus on improving the quality of care delivered.

Recommendations for policy makers include adopting these principles and strategies using a platform-wide approach. Policy makers need to engage with a wide range of stakeholders in this process and make use of the best available evidence in a transparent manner.
